# Antenatal Depression in Anuradhapura, Sri Lanka and the Factor Structure of the Sinhalese Version of Edinburgh Post Partum Depression Scale among Pregnant Women

**DOI:** 10.1371/journal.pone.0069708

**Published:** 2013-07-26

**Authors:** Suneth Buddhika Agampodi, Thilini Chanchala Agampodi

**Affiliations:** Maternal and Child Health Research Unit, Department of Community Medicine, Faculty of Medicine and Allied sciences, Rajarata University of Sri Lanka, Saliyapura, Sri Lanka; University of Pennsylvania, United States of America

## Abstract

**Background:**

Mental health problems among women of reproductive age group contribute to 7% of Global Burden of Diseases of women of all ages. Purpose of this study was to determine the prevalence and correlates of antenatal depression among pregnant women in Anuradhapura, Sri Lanka, and to explore the factor structure of EPDS.

**Methods:**

Pregnant women with gestational age of 24–36 weeks and residing in Anuradhapura district, Sri Lanka were recruited to the study using a two stage cluster sampling procedure. Sinhalese version of Edinburgh Post Partum Depression Scale (EPDS) and an interviewer administered questionnaire was use to collect data. A cut off value of 9 was used for the Sinhalese version of EPDS.

**Results:**

A total of 376 pregnant women were studied. Median EPDS score among pregnant women was 5 (IQR 2–8). Prevalence of antenatal depression in this study sample was 16.2% (n = 61). Thought of self harming (item number 10) was reported by 26 pregnant women (6.9%). None of the socio-demographic factors were associated with depression in this study sample. Having heart burn was significantly associated with depressive symptoms (p = 0.041). Sri Lankan version of EPDS showed a two factor solution. Anxiety was not emerged as a separate factor in this analysis.

**Conclusions:**

Prevalence of antenatal depression in Anuradhapura, Sri Lanka was relatively low. Anxiety was not emerged as a separate factor in the Sinhalese version of the EPDS.

## Introduction

Mental health problems of women in child bearing age is increasingly being identified as a major public health problem in both developed and under developed countries. Maternal mental health problems stands unique to other maternal morbidities as it directly hampers the psychological support and protection offered by a mother to her child, family and the community. It is meant to be a tragedy which creates a vicious cycle of complex social problems in the family context that result in life long suffering of individuals.

Mental health problems among women of reproductive age group (15–45 yrs) contributes to 7% of Global Burden of Diseases of women of all ages [1]. Estimated prevalence of perinatal mental health problems is 10–15% in developed countries. In developing countries, the prevalence is much higher, ranging from 10–41% during antenatal period [2]. The variability is thus explained by various socio-cultural and biological factors including the methods used to obtain their presence. However, perinatal mental health problems are often underestimated and undertreated in developing countries.

Among the mental health disorders related to pregnancy, depression and anxiety are the commonest. The most recent review done on perinatal depression among Asian women indicates a prevalence of anxiety and depression among 20% of pregnant women [3]. Many studies all around the world indicate the association of antenatal depression with re-occurrence of post partum depression. A long term follow up study done by Rahman et al shows high rate of persistent depression in the first year of delivery in mothers who had antenatal depression [4]. Antenatal anxiety has gained much interest during the past few decades with regard to its effect on fetus as well as a predictor of post partum depression. The AVON longitudinal study which was performed using a large cohort of mothers indicates that 14.6% of mothers having elevated levels of anxiety at least ones during pregnancy [5].

Risk factors of maternal mental health problems are found to be multi factorial including biological, psychological and various social determinants [2]. Effects of antenatal depression and anxiety have been well studied in animals and up to a certain extent among humans especially in developed countries, showing their association to preterm labor, low birth weight, post partum depression and behavioral/emotional problems of offspring. There is a growing body of evidence showing the important relationship between antenatal psychology and nutritional status of the infant in developing countries which may be a cornerstone of addressing the stagnant nutritional indicators of these countries in the future.

Sri Lanka, being a developing country in the South East Asian region has been exemplary on maternal and child care indicators over the past decades. In 2009, Sri Lanka reported a maternal mortality ratio of 34 per 100,000 live births, which is achievement of millennium development goals few years earlier than expected. The maternal health services in the country are now aiming on further reduction of maternal mortality by improving prevention, early detection and appropriate management of maternal morbidities. Despite having a very good public health system that successfully incorporates many evidence based interventions concerning maternal and child health, the component of mental health remains as an area of solicit with minimum penetration into field health services. Post partum depression is reported as a major concern and the revised pregnancy care programme of Sri Lanka (implementation in progress) included the screening for postpartum depression. However, antenatal depression has not been taken up as a major issue among policy makers as well as researchers. There are no published data available on antenatal depression in Sri Lanka. Purpose of the present study was to determine the prevalence and correlates of antenatal depression among a group of pregnant women in Anuradhapura Sri Lanka and to explore the factor structure of Edinburgh Postnatal Depression Scale (EPDS) among Sri Lankan pregnant women.

## Methods

### Ethics statement

Ethical clearance for the present study was obtained under the study on “Disease burden and economic impact of maternal morbidity”, from the Ethical Review Board of the Faculty of Medicine and Allied Sciences, Rajarata University of Sri Lanka. Informed written consent was obtained from all pregnant women prior to data collection.

### Study setting

A cross sectional descriptive study was carried out in the Anuradhapura district, Sri Lanka. Annually, around 19,000 pregnant women are registered at antenatal clinics in the Anuradhapura district which has 19 public health divisions, known as Medical Officer of Health (MOH) areas. Each MOH area is divided into sub-divisions called Public Health Midwife (PHM) areas with a population ranging from 1,500 to 3,000 in each area and the maternal health services are provided through the area PHM.

### Participants

The study population included pregnant women with a gestational age ranged from 24 to 36 weeks, and residing in the Anuradhapura district. Sample was selected from those who registered in the field antenatal clinics. We exclude pregnant women with a gestational period >36 weeks to exclude specific problems during perinatal period. As this study was a part of a larger maternal morbidity estimate study [6], only the pregnant women >24 weeks of gestation were included. A two stage cluster sampling procedure was used to recruit pregnant women to this study. In the first stage, five MOH areas were selected purposely to represent different geographical areas of the Anuradhapura district. In the second stage, eligible pregnant women within the specified gestational ages were selected using pregnant women registers available in the PHMs offices of the selected MOH areas. All those eligible women in selected MOH divisions during the study period were invited to participate.

### Study size

Based on the Sri Lankan data on postpartum depression, we hypothesized that in our population at least 30% of pregnant women would experience depressive symptoms during pregnancy. Sample size was calculated to detect at least 30% of depressive symptoms for a population of 15,000 deliveries, with 5% absolute precision and 95% confidence limits. With a 1.2 design effect for cluster design, required sample size for the present study was estimated at 384 pregnant women.

### Study tool

EPDS is a 10-item self- report scale consisting of statements describing depressive symptoms. This is a simple and well accepted assessment tool that is easy to fill in and does not require specialized psychiatric expertise for interpretation. Although it was originally developed to detect postpartum depression, it has shown as a valid and reliable tool for screening depressive symptoms during pregnancy [7], [8], [9], [10], [11]. The original questionnaire had a conceptual one factor model to detect depressive symptoms. Later, the exploratory and confirmatory factor analysis procedures using different translations of EPDS explained that in most settings this tool detects two domains; depression and anxiety [12], [13]. In some models, it showed three factor solution with question number one and two as a different domain [14], [15], [16]. EPDS is translated and validated in Sri Lanka as a part of doctoral study and tested among a large scale study in Puttlum district in 2004. This validation study proposed a cut off value of 9 for antenatal women [17]. Subsequently, this validated version of EPDS has been used in several local research (mostly unpublished), including a national survey [18]. After evaluation by the expert committee for national pregnancy care programme, screening using this validated version of EPDS is included as a part of routine post partum care since 2012. However, factor structure of EPDS has not been reported previously in Sri Lanka.

To study the association between antenatal morbidities and depression, morbidity data were obtained using a culturally adapted version of IMMPACT tool kit for productivity cost [6]. Any morbidity condition reported as affecting their day to day life and limiting daily work were included as morbidity conditions in this study.

### Procedure

All eligible pregnant women were invited to participate in the study by PHMs. Pregnant women who consented to participate in the study, were interviewed at clinic centers or sample collection centers. Trained interviewers distributed the questionnaire among pregnant women and collected data under the supervision of investigators.

### Statistical analysis

Prevalence of depressive symptoms were calculated as percentages and 95% CI for percentage. Cut off value was set at 9 based on the validation study. Distributions of depressive symptoms were compared across selected socio-demographic/economic factors and morbidity conditions using chi-square statistics. To examine the factor structure of Sinhalese version of EPDS, a principal component analysis was performed using varimax rotation.

## Results

### Study sample

After excluding 32 incomplete questionnaires, a total of 376 pregnant women participated in this study. Mean age of the study sample was 27 years (SD 5 years). Primi para women accounted for 41.5% (n = 156) of the study population. Half (50.8%) the study population was living in a nuclear family (Table 1).

**Table 1:** Characteristics of the study sample.

	n	%		
**Age group**				
<20 years	33	8.8		
20–34 years	311	82.7		
>34 years	32	8.5		
**Parity**				
First	156	41.5		
Second	112	29.8		
Third or more	108	28.7		
**Ethnicity**				
Sinhalese	352	93.6		
Tamil	3	.8		
Moor	21	5.6		
**Family type**				
Nuclear	191	50.8		
Extended	185	49.2		
**Main economic activity of the family**				
Professionals/business/administrative	25	6.6		
Other professionals/technical	47	12.5		
Security services	102	27.1		
Skilled manual workers	121	32.2		
Unskilled manual workers	81	21.5		
**Highest level of education**				
Up to grade 5	12		3.2	
Up to grade 10	170		45.2	
Passed O/L	103		27.4	
Passed A/L	71		18.9	
University degree	18		4.8	
**Per-capita income**				
<$1	34		9.0	
$1–$2	99		26.3	
>$2	224		59.6	

### Depressive symptoms

**Figure 1:**
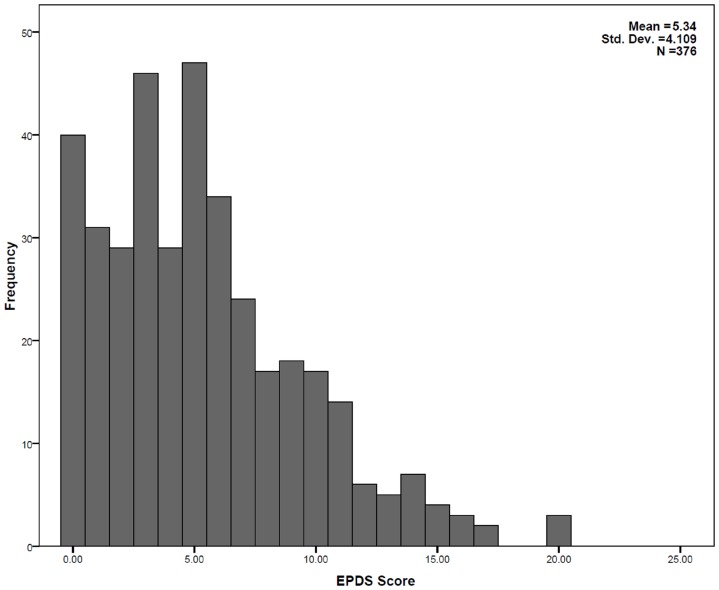
Distribution of Edinburgh Postpartum Depression Scale scores among 376 pregnant women in Anuradhapura, Sri Lanka.

Distribution of EPDS scores were skewed to right (Figure 1). Median score was 5 (IQR 2–8). Of the 376 pregnant women studied, 61(16.2%) had scores more than 9. Thought of self harming (item number 10) was reported by 26 pregnant women (6.9%).

#### Depressive symptoms and socio-demographic factors

We examined the distribution of depressive symptoms by socio-demographic and economic factors (Table 2). Distribution of depressive symptoms did not show statistically significant differences across these factors (Chi-square test, p>.05 for all factors, even after converting to binary variables).

**Table 2:** Distribution of depressive symptoms by socio-demographic and economic characteristics.

	EPDS>9		EPDS≤9	
	n	%	n	%
**Age group**				
<20 years	7	21.2	26	78.8
20–34 years	49	15.8	262	84.2
>34 years	5	15.6	27	84.4
**Parity**				
First	24	15.4	132	84.6
Second	14	12.5	98	87.5
Third or more	23	21.3	85	78.7
**Ethnicity**				
Sinhalese	54	15.3	298	84.7
Tamil/Moor	7	29.2	17	70.8
**Family type**				
Nuclear	26	13.6	165	86.4
Extended	35	18.9	150	81.1
**Main economic activity of the family**				
Professionals/business/administrative	3	12.0	22	88.0
Other professionals/technical	7	14.9	40	85.1
Security services	18	17.6	84	82.4
Skilled manual workers	21	17.4	100	82.6
Unskilled manual workers	12	14.8	69	85.2
**Highest level of education**				
Up to grade 5	3	25.0	9	75.0
Up to grade 10	27	15.9	143	84.1
Passed O/L	18	17.5	85	82.5
Passed A/L	10	14.1	61	85.9
University degree	3	16.7	15	83.3
**Per-capita income**				
<$1	8	23.5	26	76.5
$1–$2	15	15.2	84	84.8
>$2	34	15.2	190	84.8

Distribution of EPDS score by none of the factors were significant (test statistics not shown).

#### Depressive symptoms and antenatal morbidities

Antenatal morbidities affecting day today lives of pregnant women were analyzed to determine any association between these morbidities and depressive symptoms (Table 3). Of the seven leading morbidity conditions analyzed, having severe heartburning was associated with depressive symptoms (p = .041) and none of the other leading maternal morbidities were associated with antenatal depression. Of the 91 pregnant women reported as having heart burn, 21 (23.1%) had depressive symptoms compared to 40 (14%) among those who were not having heart burn (n = 285).

**Table 3:** Distribution of depressive symptoms by antenatal morbidities.

	EPDS score				Significance	
	>9		≤9			
	n	%	n	%		
**Nausea and vomiting**						
Present	43	16.3%	221	83.7%	?^2^	0.003
Absent	18	16.1%	94	83.9%	p	0.985
Backache						
Present	18	15.8%	96	84.2%	?^2^	0.023
Absent	43	16.4%	219	83.6%	p	0.880
Cramps						
Present	12	16.4%	61	83.6%	?^2^	.003
Absent	49	16.2%	254	83.8%	p	0.956
**Heartburn/regurgitation**						
Present	21	23.1%	70	76.9%	?^2^	4.149*
Absent	40	14.0%	245	86.0%	p	0.041
Headache						
Present	10	17.2%	48	82.8%	?^2^	0.051
Absent	51	16.0%	267	84.0%	p	0.819
**Tiredness**						
Present	10	20.4%	39	79.6%	?^2^	0.726
Absent	51	15.6%	276	84.4%	p	0.394
**Vertigo**						
Present	19	22.6%	65	77.4%	?^2^	3.255
Absent	42	14.4%	250	85.6%	p	0.071

* significant.

### Factor structure of Sinhalese version of EPSD

No published literature is available for antenatal depression in Sri Lanka except the validation study. Thus the construct validity of EPDS has not been studied previously. We analyzed the construct validity of the Sinhalese version of EPDS, using the principal component analysis option of SPSS.

Two orthogonal factors with Eigenvalues >1 were obtained after varimax rotation. The factor loadings of each item are shown in Table 4. The first factor included all items except items 1 and 2 which explore anhedonia. Item 8 (“I have felt sad or miserable”) loaded in both factors. The Kaiser–Meyer–Olkin measure for the data was 0.801, which suggested that these data were suitable for factor analysis, as the measure exceeded the recommended value of 0.60. Bartlett's test of sphericity reached statistical significance (p<0.001), which supported the factorability of the correlation matrix. A two-factor solution was found to explain 42.42% of the total variance across all items. Factor 1, accounted for 29.57% of the variance and Factor 2, accounted for 12.83%.

**Table 4:** Rotated Component Matrix of Sinhalese version of EPDS for pregnant women.

	Component	
	1	2
**Q5**	.671	
**Q9**	.613	
**Q4**	.598	
**Q3**	.589	
**Q7**	.544	
**Q10**	.36	
**Q6**	.424	
**Q2**		.778
**Q1**		.777
**Q8**	.439	.470

Extraction Method: Principal Component Analysis.

Rotation Method: Varimax with Kaiser Normalization.

Rotation converged in 3 iterations.

## Discussion

WHO estimates depressive disorders to be the second leading cause of global disease burden by 2020 [19]. Depressive disorders and other mental health illnesses will have a more profound effect on pregnant women in developing countries due to present lack of focus on maternal mental health. Maternal health experts in developing countries are concentrating on direct causes of maternal deaths, understandably due to the high maternal mortality ratios in those countries. With this background, depression has not been given a priority, especially during the pregnancy. In Sri Lanka, maternal mental wellbeing is included as a part of the pregnancy care programme. However, focused interventions and screening for detection of antenatal mental health problems are still not in practice. Published literature is not available pertaining to antenatal depression in Sri Lanka. The present study shows that the prevalence of antenatal depression is 16.2% (95% CI 12.8–20.2%) in Anuradhapura district, Sri Lanka.

Reported prevalence of antenatal depression in developing countries in the region, shows a wide range of values. A community based study from Bangladesh reported a prevalence of 33% [20]. In Pakistan, several studies reported prevalence estimates ranging from 25%–48% [21], [22]. Among Asian women in UK, prevalence was reported as 30.7% [23]. Reports from East Asian countries like China (4.4%) [24] and Japan shows a relatively low prevalence of antenatal depression. Even in Western hemisphere, most of the reported prevalence rates are higher than the Sri Lankan prevalence value we observed. This relatively low prevalence could be partly due to extensive family support, value placed on pregnant mothers and the socio-cultural support for pregnant women in Sri Lankan society. However, this low prevalence in pregnancy period could not be attributed directly to those reasons mentioned, because the postpartum depression in Sri Lanka is high. In 2010, we estimated postnatal depression among a national representative sample, which showed a prevalence of 27.1% with 2.9% suicide ideation. Further studies are required to investigate whether this antenatal depression prevalence value could be generalized to Sri Lanka. If so, factors that specifically prevent antenatal depression should be investigated further in order to apply this knowledge to postpartum mothers.

Our observation on lack of association of antenatal depression and socio-demographic and economic factors are slightly different from the previous studies. Previous studies has shown most of the pregnancy related and socio-demographic variables such as income [22], [25], [26], [27], age [28] and minor ailments of pregnancy [29] as risk factors for antenatal depression. Even our previous study on postpartum depression in Sri Lanka showed significant association with parity and income [18]. Nevertheless, sample size for this study was calculated for prevalence estimates and lack of association could have been due to lack of power to detect any.

Another concern about the study finding is the results of factor analysis. Factor structure analysis of EPDS among antenatal women in recent past clearly showed the anxiety component within the tool, either as three factor [14], [15], [30] or two factor [12], [13], [31] model. Our study was unable to show this structure among pregnant mothers. Items 3 to 7 and 9–10 loaded primarily and exclusively to a single factor which included both depressive and anxiety domains. Factor 1 and 2 (items exploring anhedonia) exclusively loaded in to a single factor. Anhedonia as a single factor was observed in several versions of EPDS where three factor model is reported [14], [32]. This observation also needs further investigations in Sri Lanka.

Interpretation of this study should be done within the limitations of study design. The original sample size calculation was done to detect the estimated prevalence. Ideally sample size should have been increased to analyzed factors affecting antenatal depression. The sampling procedure was done by inviting pregnant mothers at household level to participate in the study. However, data collection was carried out in the clinics and investigation centre. There is a possibility that women who are depressed have refused to participate, which ends up with a selection bias. This selection bias could under estimate the prevalence of antenatal depression.

In conclusion, our study shows that the prevalence of antenatal depression in Anuradhapura, Sri Lanka is relatively low and we were unable to show any association between the commonly known risk factors and antenatal depression. Factor structure of the Sinhalese version of EPDS shows a two factor model in which depressive and anxiety component are represented by a single factor. We recommend further studies to explore our findings.
